# Combined inoculation with dark septate endophytes and arbuscular mycorrhizal fungi: synergistic or competitive growth effects on maize?

**DOI:** 10.1186/s12870-021-03267-0

**Published:** 2021-10-29

**Authors:** Linlin Xie, Yinli Bi, Shaopeng Ma, Jianxuan Shang, Qincheng Hu, Peter Christie

**Affiliations:** 1 State Key Laboratory of Coal Resources and Safe Mining, China University of Mining and Technology (Beijing), Beijing, 100083 China; 2 Institute of Ecological and Environmental Restoration in Mining Areas of West China, Xi’an University of Science and Technology, Xi’an, 710054 China; 3 Shaanxi Coal and Chemical Industry Group Co., Ltd, Xi’an, 710076 China

**Keywords:** Arbuscular mycorrhizal fungi, Dark septate endophytes, Maize, Inoculation

## Abstract

**Background:**

Effects on maize were assessed of dual inoculation with arbuscular mycorrhizal fungi (AMF) and dark septate endophytes (DSE) isolated from other plant species.

**Methods:**

Suspensions of DSE isolated from *Stipa krylovii* were prepared at different densities (2, 4, and 8 × 10^5^ CFU mL^− 1^) and inoculated separately (AMF or DSE) or together (AMF + DSE), to explore their effects on maize growth.

**Results:**

Inoculation with AMF or medium and high densities of DSE and combined inoculation (AMF + DSE) increased plant above-ground growth and altered root morphology. Differences in plant growth were attributable to differences in DSE density, with negative DSE inoculation responsiveness at low density. AMF promoted plant above-ground growth more than DSE and the high density of DSE promoted root development more than AMF. Combined inoculation might lead to synergistic growth effects on maize at low density of DSE and competitive effects at medium and high DSE densities.

**Conclusions:**

AMF and DSE co-colonized maize roots and they had positive effects on the host plants depending on DSE density. These findings indicate the optimum maize growth-promoting combination of AMF and DSE density and provide a foundation for further exploration of potentially synergistic mechanisms between AMF and DSE in physiological and ecological effects on host plants.

**Supplementary Information:**

The online version contains supplementary material available at 10.1186/s12870-021-03267-0.

## Background

Dependence on chemical fertilizers and pesticides results in pesticide residues in crops and soils and increases production costs. Microbial technology has been widely used to solve this problem by inoculating roots with endophytic fungi to exploit their potentially symbiotic associations and stimulate plant growth to provide pollution-free production systems.

Arbuscular mycorrhizal fungi (AMF) are important soil fungi that inhabit the roots of most terrestrial plant species with which they form potentially symbiotic associations. Under appropriate conditions the AMF can increase plant nutrient uptake and carbon fixation by photosynthesis, and enhance plant tolerance to biological or abiotic (e.g., drought, salinity, and low temperatures) stresses [1–5]. For example, the extraradical mycelium of AMF form an important bridge to transport nutrients outside the roots to the intraradical mycelium [6] and can promote an effective relationship between plants, bacteria and beneficial fungi [7]. The concentrations of glomalin in soil have often been correlated with AMF biomass and the formation, deposition, and/or decomposition of glomalin in soils seem to be largely dependent on a multitude of interactions among plants, AMF, and other soil microorganisms, including prokaryotes [8]. Simulation experiments indicate that inoculation of plants with AMF increases plant biomass and alleviates mechanical damage to the root system [9]. AMF inoculation also increases the abundance of certain beneficial bacterial species and decreases that of certain pathogenic fungi in the rhizosphere [10].

Dark septate endophytes (DSE) are a miscellaneous group of asexually propagating fungal endophytes that colonize living root tissues intracellularly and intercellularly in a range of extreme ecosystems [11, 12]. DSE have a wide host range and ecological distribution including mycorrhizal [13, 14] and non-mycorrhizal [15, 16] plants. In contrast to arbuscular mycorrhizal fungi, DSE not only grow inter- and intra-cellularly within the root cortex but can extend into the vascular tissue [17, 18]. Typical micro structures of DSE such as dark septate hyphae and microsclerotia are formed with different degrees of melanism [14, 19, 20]. DSE melanin can assist host plants in resisting adverse environmental conditions by protecting fungal mycelium from abiotic stresses such as high temperatures, drought, and potentially toxic elements [21, 22]. Numerous studies suggest that DSE can promote the uptake and transformation of mineral and organic nutrients and increase the adaptability of the host or the nutritional status of plants to increase resistance to diseases and other stresses [11, 23–26].

DSE may form a root-fungal association with the host [14], modify the mycorrhizal status of the plant, and thus modulate a different symbiotic association in the rhizosphere [27]. A previous study using DSE with other microorganisms such as *Trichoderma viride* assessed the effects of combined inoculation on plant growth and substantiated their positive influence [28].

Low colonization rates by AM fungi at the pre-symbiotic stage may be compensated by DSE by some weak competitive or antagonistic interactions between the two fungal groups [29, 30]. A succession of dominant fungal colonizers from AMF to DSE has been observed in the roots of the grass *Deschampsia flexuosa* and may be related to the different nutrient acquisition strategies of the two fungal groups [31]. Numerous separate studies of AMF and DSE have drawn widespread attention to their favorable ecological functions [32] but combined inoculation of plants with AMF and DSE have been little studied and remain poorly understood [33].

The objective of the present study was to obtain insights into the interactions between AMF and DSE and to extend their potential use in future field application. Suspensions of DSE isolated from *Stipa krylovii* were prepared at different densities and AMF and DSE were used to inoculate maize separately (AMF or DSE) and together (AMF + DSE) to investigate their effects on the host plant. The work addressed the questions of whether DSE and AMF can co-colonize maize roots in vitro  and whether inoculation with DSE and AMF has synergetic or competitive effects on the growth of the host plant?

## Results

### Plant growth

Inoculation with AMF and DSE had significant effects on plant growth (Table 1). Plant height, ground diameter, and leaf area in combined treatments AM + MD (64.4 cm) and AM + HD (65.6 cm) were higher than with separate inoculants and the uninoculated control. Shoot fresh/dried biomass in treatment AM + HD were significantly higher than in other treatments (*P* < 0.05). Root fresh and dried biomass in all combined inoculation treatments (AM + LD, AM + MD, AM + HD) were significantly higher than in separate inoculation treatments and the control (*P* < 0.05). The maximum values overall occurred in treatment AM + HD. Moreover, the mean inoculation responsiveness in separate and combined inoculation treatments was 19.3 and 32.9%, respectively. The average positive inoculation responsiveness was 57.2% (range 46.1–79.9%) with the maximum observed in AM + HD. Interestingly, the minimum plant biomass and leaf area occurred in treatment LD and was not significantly different from the control. The inoculation responsiveness in LD was − 16.2%.

**Table 1:** Effects of different treatments on maize growth and inoculation responsiveness

Treatments	Height (cm)	Ground diameter (mm)	Leaf area (cm^2^)	Shoot fresh biomass (g)	Root fresh biomass (g)	Shoot dried biomass (g)	Root dried biomass (g)	Inoculation responsiveness (%)
CK	35.47 ± 4.83b	0.54 ± 0.02d	192.72 ± 33.32d	8.89 ± 3.54c	1.7 ± 0.82e	1.32 ± 0.39c	0.74 ± 0.29d	None
AM	63.8 ± 4.62a	1.07 ± 0.08ab	767.67 ± 107.50abc	30.68 ± 3.88ab	8.05 ± 0.51bc	4.11 ± 0.70ab	2.92 ± 0.39abc	70.72
LD	39.97 ± 4.29b	0.56 ± 0.02d	176.4 ± 27.44d	6.38 ± 1.90c	1.26 ± 0.43e	1.06 ± 0.31c	0.72 ± 0.12d	−16.16
MD	45 ± 4.50b	0.75 ± 0.05c	283.28 ± 27.99d	10.31 ± 1.94c	3.94 ± 0.82d	1.99 ± 0.30bc	1.83 ± 0.56 cd	46.07
HD	59.1 ± 4.76a	0.99 ± 0.01b	644.19 ± 63.73bc	23.71 ± 4.05b	6.22 ± 0.13c	3.86 ± 0.33b	2.63 ± 0.30bc	68.28
AM + LD	61.33 ± 5.69a	1.06 ± 0.07ab	635.04 ± 52.19c	25.89 ± 2.35b	8.92 ± 0.85ab	4.11 ± 0.98ab	3.38 ± 0.63ab	72.50
AM + MD	64.37 ± 2.76a	1.08 ± 0.03ab	789.88 ± 8.98ab	28.06 ± 1.19ab	10.67 ± 0.31a	3.81 ± 0.42b	3.65 ± 0.50ab	72.37
AM + HD	65.63 ± 5.28a	1.16 ± 0.11a	873.51 ± 45.56a	35.38 ± 4.97a	10.78 ± 0.98a	6.12 ± 1.59a	4.15 ± 0.79a	79.94

CK, treatments with sterilized DSE and AMF; AM, sterilized DSE with AMF; LD, low concentration of DSE with sterilized AMF; MD, medium concentration of DSE with sterilized AMF; HD, high concentration of DSE with sterilized AMF. AM + LD → AM + HD, different concentration of DSE and AMF. Data followed by different letters in the same column are significantly different at *P* < 0.05

**Fig. 1:**
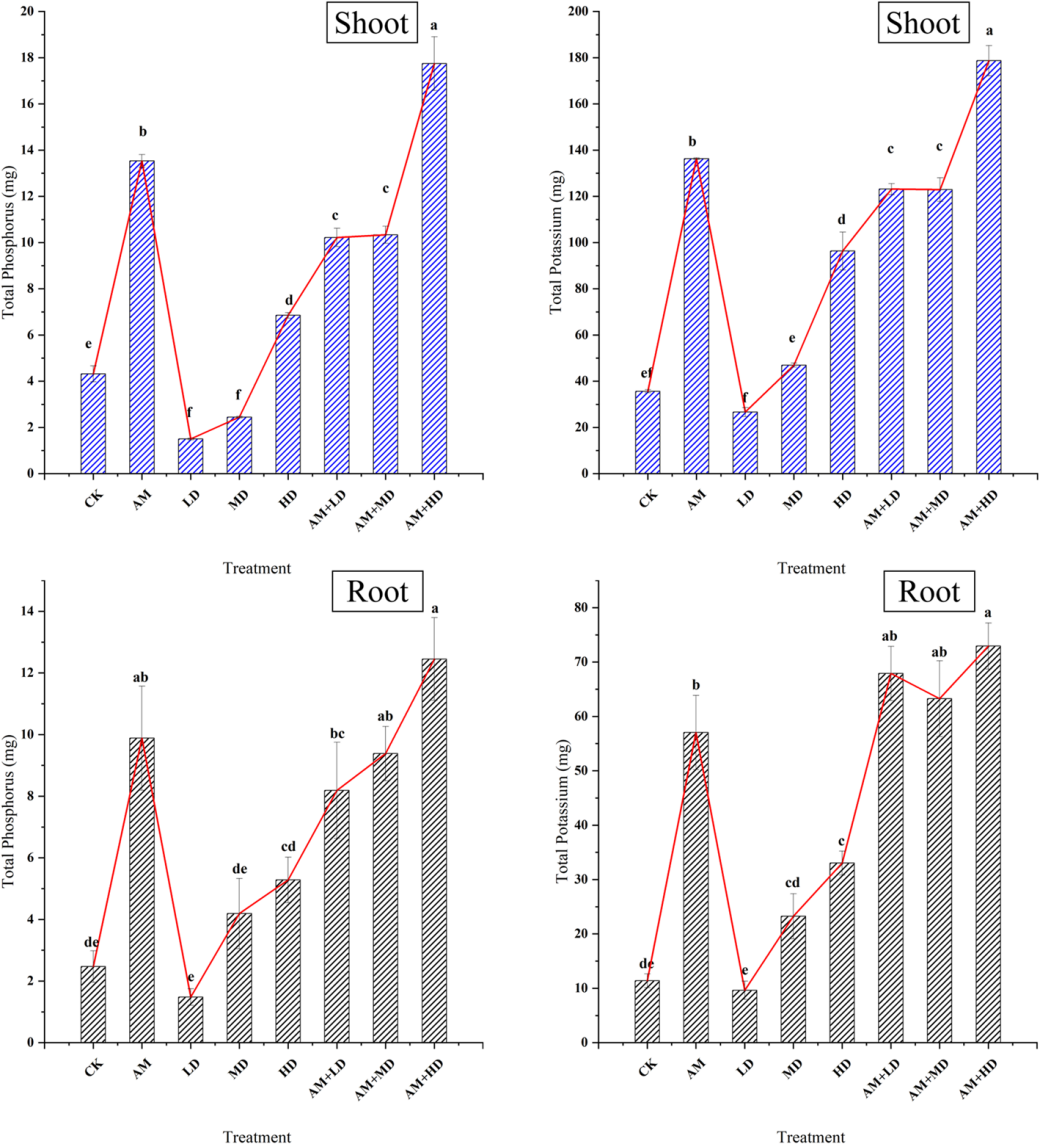
Effects of the different treatments on plant total phosphorus (TP) and total potassium (TK). **A**, shoots; **B**: roots. CK: controls with sterilized DSE and/or AMF; AM, sterilized DSE with AMF; LD, low concentration of DSE with sterilized AMF; MD, medium concentration of DSE with sterilized AMF; HD, high concentration of DSE with sterilized AMF; AM + LD, low concentration of DSE with AMF; AM + MD, medium concentration of DSE with AMF; AM + HD, high concentration of DSE with AMF

Shoot total P and K concentrations were also significantly increased by AMF inoculation (Fig. 1). Plant TP and TK increased significantly with increasing DSE density with a minimum value in LD, and combined inoculation with AMF showed similar results with a maximum value in AM + HD.

### Root system morphology and fungal colonization

Root system morphology was significantly affected by the inoculation treatments (Table 2). Separate and combined inoculation significantly increased total root length, root surface area and total root volume except in separate treatment LD (*P* < 0.05), with maximum inoculation responsiveness observed in AM + HD (63.3, 54.3 and 70.2%, respectively). The root mean diameter in all inoculation treatments was significantly larger than in the control (*P* < 0.05). Interestingly, the root morphology inoculation responsiveness of LD was negative.

**Table 2:** Effects of different treatments on root morphological traits and inoculation responsiveness of maize

Treatments	Total Length (cm)	Inoculation responsiveness (%)	Surf Area (cm2)	Inoculation responsiveness (%)	Avg Diam (mm)	Inoculation responsiveness (%)	Root Volume (cm3)	Inoculation responsiveness (%)
CK	1357.08 ± 90.4 cd	0	966.68 ± 41.55b	0	1.27 ± 0.13b	0	30.27 ± 1.81c	0
AM	1932.01 ± 117.87bc	30	1680.12 ± 109.32a	42.46	1.54 ± 0.06b	17.53	35.76 ± 1.87c	15.35
LD	1072.06 ± 48.85d	−26.59	923.38 ± 15.34b	−5	1.51 ± 0.18b	15.89	29.22 ± 2.05c	−3.59
MD	1991.39 ± 321.94bc	31.85	1619.53 ± 121.46a	40.31	1.59 ± 0.07ab	20.13	35.23 ± 1.91c	14.08
HD	2610.07 ± 116.98b	48.01	1687.04 ± 113.26a	42.7	1.61 ± 0.07ab	21.12	41.21 ± 0.68c	26.55
AM + LD	2189.76 ± 182.43bc	38.03	1828.51 ± 78.34a	47.13	1.53 ± 0.07b	16.99	65.62 ± 3.8b	53.87
AM + MD	2536.56 ± 134.44b	46.5	1873.19 ± 185.75a	48.39	1.71 ± 0.15ab	25.73	78.14 ± 4.06b	61.26
AM + HD	3701.72 ± 363.28a	63.34	2114.4 ± 154.32a	54.28	2.1 ± 0.18a	39.52	101.58 ± 10.07a	70.2

CK, treatments with sterilized DSE and AMF; AM, sterilized DSE with AMF; LD, low concentration of DSE with sterilized AMF; MD, medium concentration of DSE with sterilized AMF; HD, high concentration of DSE with sterilized AMF. AM + LD → AM + HD, different concentration of DSE and AMF. Data followed by different letters in the same column are significantly different at *P* < 0.05.

**Fig. 2:**
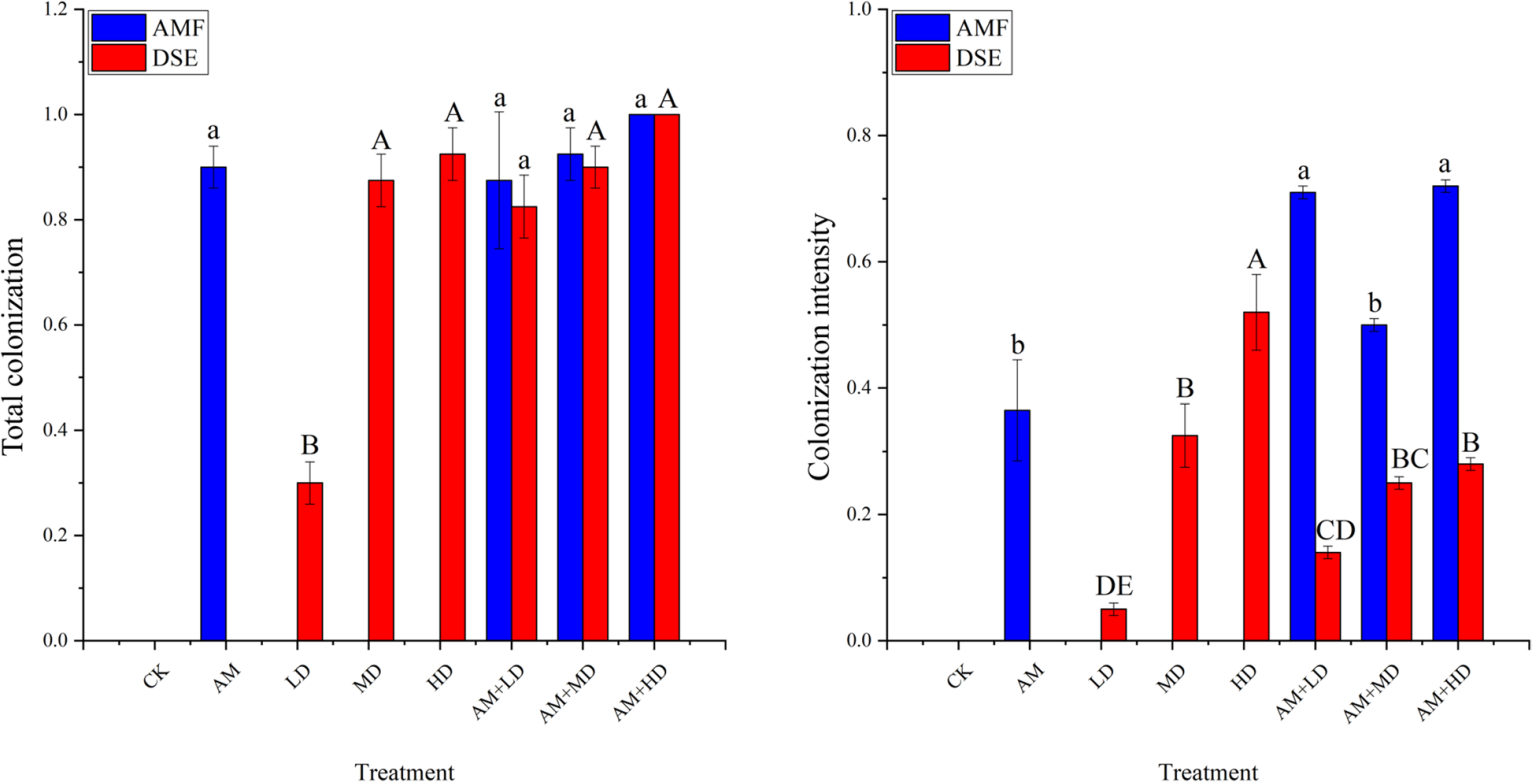
Effects of different treatments on AMF and DSE colonization. CK, treatments with sterilized DSE and AMF; AM, sterilized DSE with AMF; LD, low concentration of DSE with sterilized AMF; MD, medium concentration of DSE with sterilized AMF; HD, high concentration of DSE with sterilized AMF. AM + LD → AM + HD, different concentration of DSE and AMF. Different letters above the columns are significantly different at *P* < 0.05

Roots in the inoculation treatments were highly colonized with AMF and DSE but no colonization was observed in control roots (Fig. 2). The total colonization and colonization intensity of AMF and DSE were significantly different in the different inoculation treatments. With DSE inoculation alone the maximum values occurred in treatment HD. Total colonization and colonization intensity of AMF increased in AMF + DSE combined inoculation compared with AMF inoculation alone and DSE total colonization also increased compared with DSE inoculation alone. DSE colonization intensity was higher in treatment AM + LD than in LD treatment and lower in AM + MD and AM + HD than in the corresponding separate inoculation treatments.

### Photosynthesis and leaf chlorophyll content

**Fig. 3:**
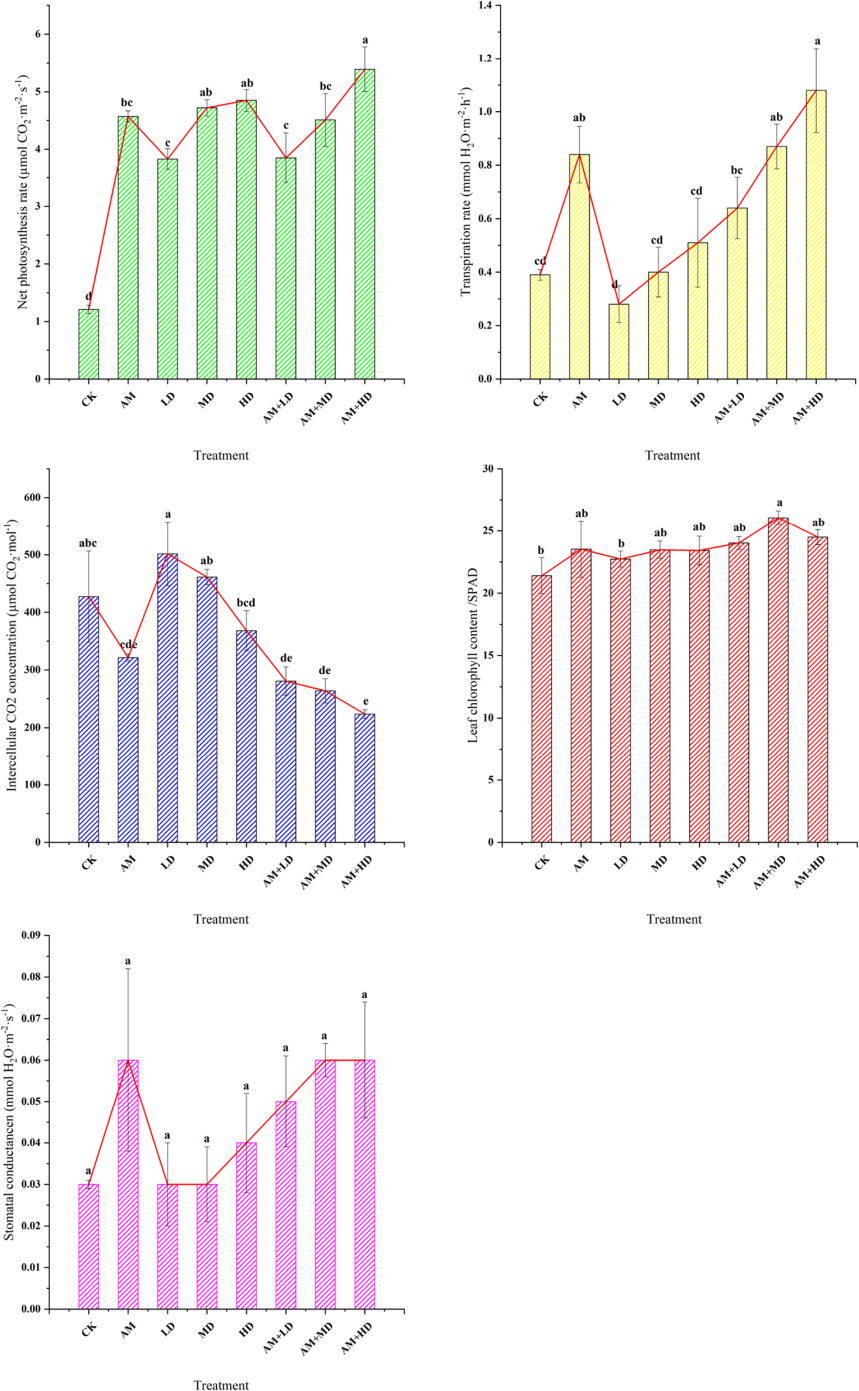
Changes in plant photosynthesis and leaf chlorophyll content. CK, treatments with sterilized DSE and AMF; AM, sterilized DSE with AMF; LD, low concentration of DSE with sterilized AMF; MD, medium concentration of DSE with sterilized AMF; HD, high concentration of DSE with sterilized AMF. AM + LD → AM + HD, different concentration of DSE and AMF. Different letters above the columns are significantly different at *P* < 0.05

Photosynthesis was significantly greater with AMF and DSE inoculation. Net photosynthetic rate and transpiration rate were significantly higher in treatment AM + HD than in the other treatments (Fig. 3, *P <* 0.05). The maximum and minimum intercellular CO_2_ concentrations were observed in LD (502 μmol CO_2_ mol^− 1^) and AM + HD (223 μmol CO_2_ mol^− 1^). Stomatal conductance was not significantly different between the separate treatments and the combined treatments and control, with the maximum observed in treatments AM, AM + MD and AM + HD. Plant leaf chlorophyll content ranged from 21.4 to 26.1 and followed the sequence: AM + MD > AM + HD > AM + LD > AM > MD > HD > LD > CK.

### Plant endogenous hormones

**Fig. 4:**
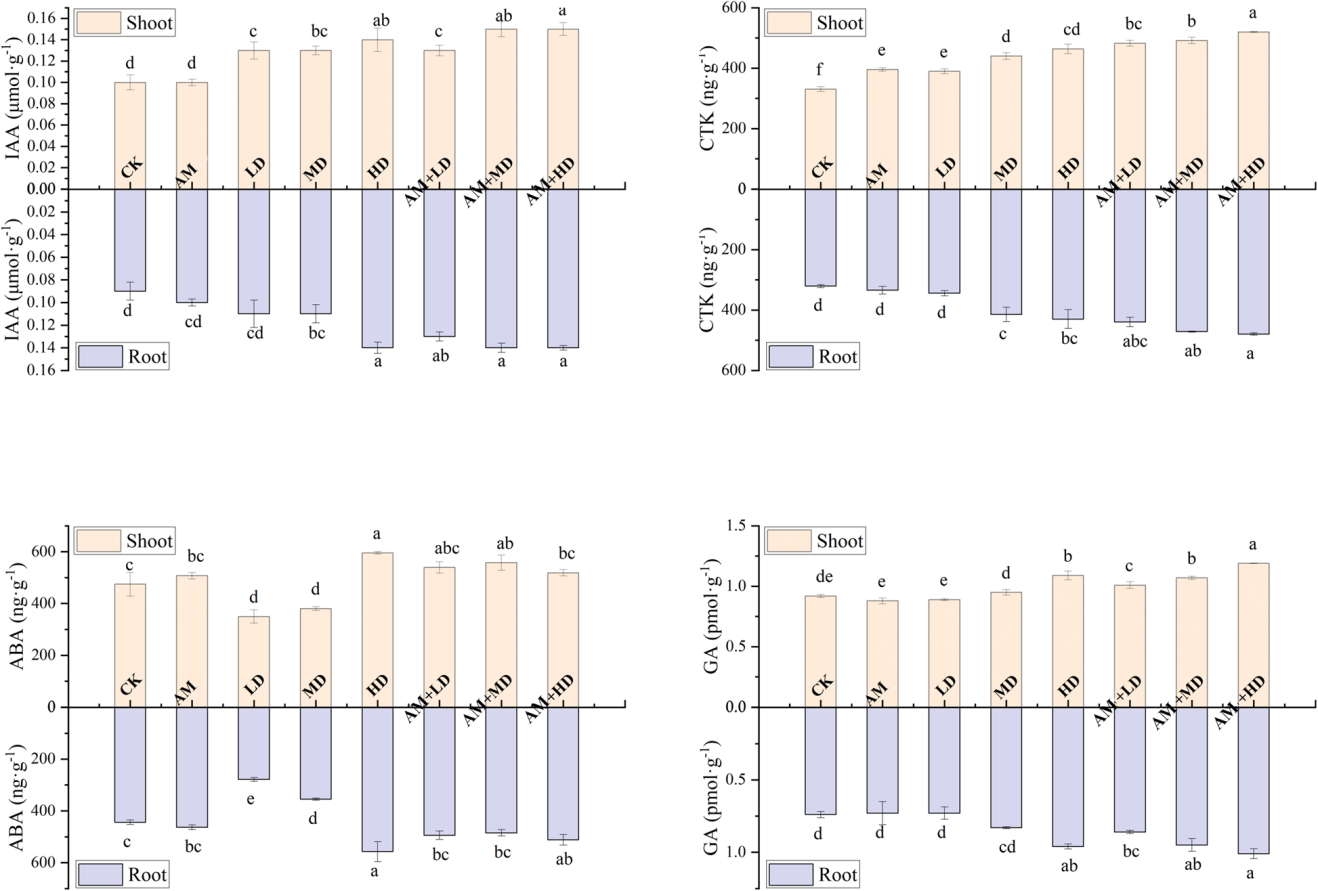
Effects of fungal inoculation on IAA, GA, CTK and ABA levels in maize roots and shoots. CK, controls with sterilized DSE and/or AMF; AM, sterilized DSE with AMF; LD, low concentration of DSE with sterilized AMF; MD, medium concentration of DSE with sterilized AMF; HD, high concentration of DSE with sterilized AMF. AM + LD → AM + HD, different concentrations of DSE and AMF. Different letters above the columns indicate significant differences at *P* < 0.05

Inoculation with AMF and DSE significantly affected the endogenous hormones (IAA, CTK, GA, and ABA) of the shoots and roots (Fig. 4). AMF inoculation significantly increased root CTK accumulation. DSE treatment MD significantly increased CTK and IAA accumulation in the shoots and roots and reduced ABA accumulation. Treatment HD and combined inoculation (AM + LD, AM + MD, AM + HD) significantly increased the accumulation of the four endogenous hormones. The maximum IAA, CTK and GA were observed in AM + HD and the maximum and minimum ABA were observed in HD and LD in the shoots and roots.

### Soil physicochemical properties

EEG, TG and ALP were significantly lower in the control than in the inoculated treatments (Table 3). DSE inoculation significantly increased EEG, TG and ALP by 1.26, 0.39 and 16.1%, respectively, under separate inoculation and by 2.86, 1.35 and 8.6%, respectively, in the combined inoculation treatments. The maximum EEG and TG were observed in AM + HD and the maximum AP, AK and ALP in LD. Inoculation with AMF and DSE significantly reduced the activity of soil urease.

**Table 3:** Edaphic variables under different treatments

Treatments	AP (mg/kg)	AK (mg/kg)	TN (mg/kg)	EEG (μg /g)	TG (μg /g)	ALP (μg/g/h)	U (μg/g/h)
CK	2.84 ± 0.17ab	124.43 ± 2.87abc	11.51 ± 0.25bc	99.47 ± 0.29b	626.21 ± 2.3c	53.22 ± 0.91b	13.22a
AM	2.94 ± 0.14ab	114.03 ± 1.42 cd	11.27 ± 0.49c	100.45 ± 0.95ab	625.76 ± 1.29c	60.07 ± 1.62ab	12.81 ± 0.65a
LD	3.65 ± 0.2a	132.18 ± 3.92a	12.82 ± 0.21ab	100.40 ± 0.21ab	627.12 ± 0.92bc	63.97 ± 2.32a	10.11 ± 0.69b
MD	2.91 ± 0.16ab	122.57 ± 1.52abc	11.76 ± 0.27bc	101.07 ± 0.28ab	627.5 ± 1.94bc	63.43 ± 2.04a	9.13 ± 0.29bc
HD	3.14 ± 0.56ab	112.60 ± 3.1 cd	12.43 ± 0.09abc	100.75 ± 0.15ab	631.35 ± 0.76abc	62.82 ± 3.89ab	7.98 ± 0.32bc
AM + LD	2.34 ± 0.13b	129.32 ± 2.01ab	12.75 ± 0.24ab	100.99 ± 0.58ab	630.11 ± 2.68abc	59.37 ± 0.99ab	7.65 ± 0.55c
AM + MD	2.53 ± 0.01ab	117.33 ± 2.43bcd	13.59 ± 0.41a	103.08 ± 0.96a	636.32 ± 2.63ab	57.27 ± 1.85ab	7.54 ± 0.11c
AM + HD	2.59 ± 0.42ab	108.2 ± 0.98d	12.25 ± 0.01abc	103.14 ± 0.24a	637.81 ± 1.79a	57.98 ± 1.89ab	7.21 ± 0.22c

*AP* Available phosphorus, *AK* Available potassium, *TN* Total nitrogen, *EEG* Easily extractable glomalin-related soil protein, *TG* Total glomalin-related soil protein, *ALP* Activity of alkaline phosphatase, *U* Activity of soil urease. CK, treatments with sterilized DSE and AMF; AM, sterilized DSE with AMF; LD, low concentration of DSE with sterilized AMF; MD, medium concentration of DSE with sterilized AMF; HD, high concentration of DSE with sterilized AMF. AM + LD → AM + HD. Different concentration of DSE and AMF. Data followed by different letters in the same column are significantly different at *P* < 0.05

### Correlation analysis

**Fig. 5:**
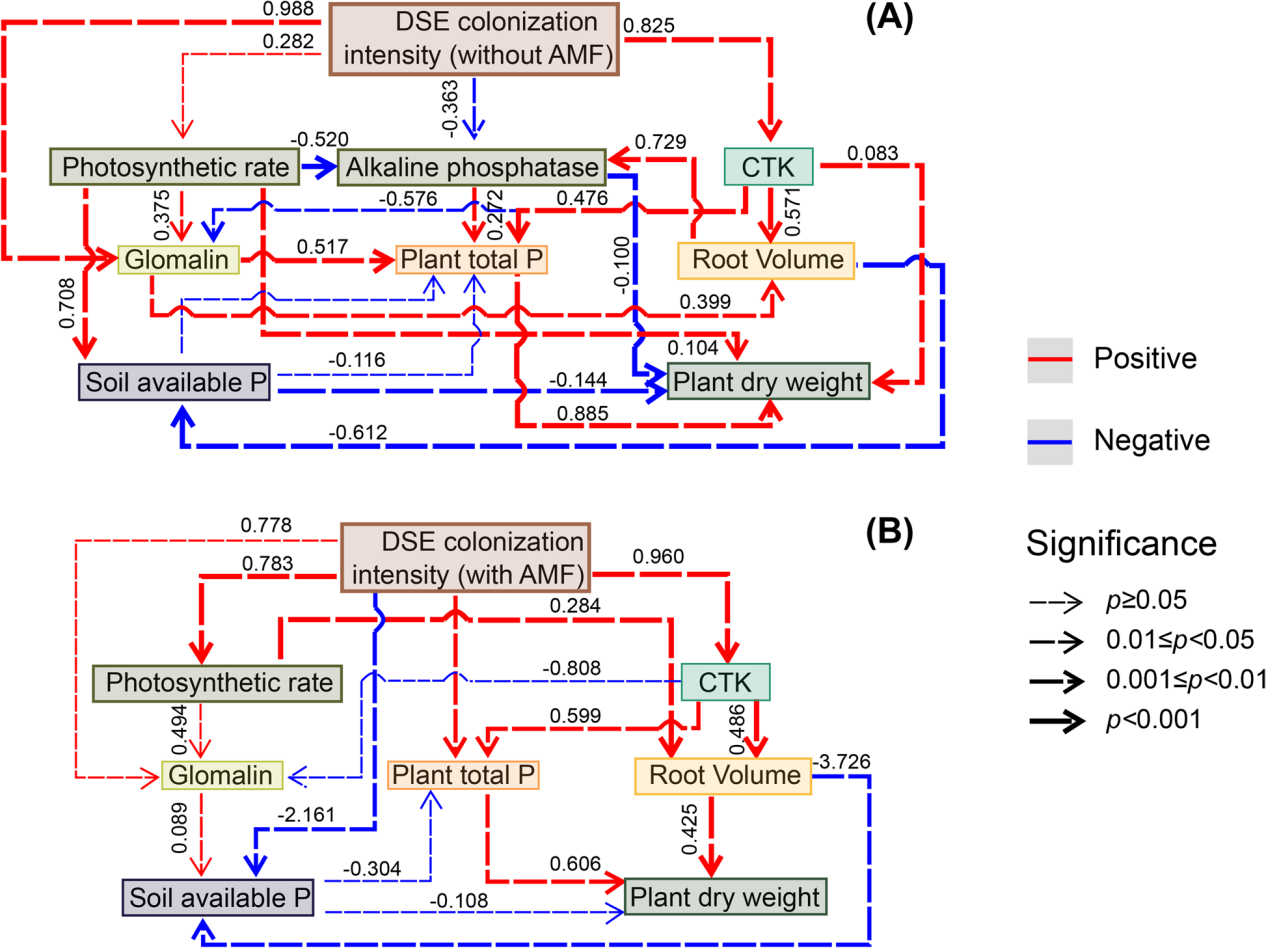
Structural equation model (SEM) showing the causal relationships among DSE colonization intensity, plant growth and soil variables. The final model fits the data well: maximum likelihood

 ﻿Pearson﻿’s correlation analysis shows significant relationships between DSE colonization intensity, plant growth and soil variables (Fig. S1). SEM was used to quantify the relative effects of DSE colonization intensity, plant CTK, photosynthetic rate, plant biomass, root volume, plant total P, soil AP, ALP and glomalin content using the correlation coefficients (*R* values). DSE infection intensity increased the accumulation of plant total P by directly increasing plant CTK content and soil glomalin, which indirectly increased plant biomass in separate inoculation treatments (χ2 = 18.329, df = 15, *P* = 0.246, RMSEA = 0.098, GFI = 0.87, AIC = 78.329; Fig. 5A). DSE infection intensity increased plant biomass indirectly by increasing plant photosynthetic rate and root volume in combined inoculation treatments (χ2 = 11.083, df = 9, *P* = 0.270, RMSEA = 0.099, GFI = 0.90, AIC = 65.083; Fig. 5B). Moreover, soil AP was negatively correlated with plant biomass and root volume in across treatments.

## Discussion

### AMF and DSE colonization

AMF are known to be influenced by the activities of other soil microorganisms and share ecological niches with DSE [27]. Previous extensive studies on the physiological and ecological functions of AMF or DSE and their respective inoculation effects have been widely reported [34–37]. However, understanding of the effects of both fungal groups colonizing plants together is lacking. Here, the effects of combining AMF and DSE at various concentrations on maize were investigated.

Typical AMF and DSE root structures were observed, indicating that AMF and DSE can grow together and colonize the roots simultaneously to form a combined potentially symbiotic structure. According to our experimental data, AMF total colonization and colonization intensity and LD colonization intensity were higher in AMF + DSE combined inoculated plants compared with separate AMF or LD inoculation. However, DSE colonization intensities in AM + MD and AM + HD were lower than in the corresponding separate DSE inoculations. We therefore speculate that AMF and low-density DSE colonization were promoted by combined inoculation but niche competition might exist between AM and MD or HD.

### Effects of AMF or DSE alone on plant growth

Separate inoculation with AMF or DSE increased maize growth (plant height, ground diameter, leaf area, plant biomass) and root system morphology (total root length, root surface area, root volume) compared to the control. DSE inoculation did not significantly affect root diameter and this is consistent with the observations of Li et al. [22]. The plant growth promoting efficacy of AMF was greater than that of DSE inoculation at all three densities but the root system morphology effects of AMF were lower than those of HD. The inoculation responsiveness of LD was negative. We suggest that AMF may significantly promote above-ground plant growth more than DSE but DSE at high density had a greater effect than AMF on root development.

Similarly, DSE inoculum at high density significantly increased shoot and root endogenous hormone levels. This supports the conclusions of Liu et al. [38] who report that DSE inoculation promoted root growth by regulating the content and percentage of endogenous hormones to resist drought stress of the host plant. Soil available P and ALP were maximum in LD inoculation but total P in roots, stems and leaves was minimum, indicating that DSE may have released more phosphatase promoting the accumulation of soil available P and supplied the nutrient to itself rather than to maize at LD [39]. Thus, the inoculum density of DSE may be a key factor determining whether the relationship between DSE and the host is mutualistic.

### Interactions between AMF and DSE in plant growth

Combined inoculation with AMF and DSE had significant direct effects on plant growth, root development, plant endogenous hormone levels, and soil properties relative to the control and separate inoculation. The inoculation responsiveness of AM + LD in terms of plant biomass, total root length, root surface area and root volume was greater than that of AM and LD summed but MD and HD showed the opposite trend. This suggests that combined inoculation might lead to synergistic plant growth effects at the low DSE density and to competitive effects at medium and high densities. This supports the hypothesis in AMF and DSE colonization. Nevertheless, AM + MD and AM + HD showed higher growth-promoting efficacies of combined inoculation.

Inoculation responsiveness values of AM + HD to plant growth, root development, plant shoot and root endogenous hormone (ABA, GA, CTK) levels, and EEG and TG in soil were 79.9, 56.8, 31.3, 3.5 and 1.8%, respectively. Correlation analysis shows that EEG and TG were positively correlated with plant growth and physiological indices. It is well known that the soil organic carbon (SOC) pool is an important regulator of carbon fluxes between the atmosphere and the biosphere. Two BRSP fractions (EEG and TG) have been widely reported to be correlated with SOC in previous studies as found in a range of environments [40–42]. The contribution of glomalin to the stocks of soil carbon has also been confirmed [8]. Here, in combined inoculation treatments DSE may have stimulated AMF to release EEG and TG and increase soil carbon for fungal growth. We suggest that the beneficial effects of AMF + DSE on plant growth might be explained by soil carbon accumulation and nutrient exchange with plants and the increases in phytohormone production that they promote [38].

## Conclusions

Here, we explore associations between AMF and DSE (derived from the roots of *Stipa krylovii*) colonizing maize. Inoculum of AMF and of three densities DSE led to the combined colonization of roots and formed a compound potentially symbiotic association. DSE inoculum at medium and high densities increased plant above-ground growth and changed root morphology and at a low density gave negative effects. The degree of DSE colonization (or the density of DSE inoculum) might be a key factor determining whether the relationship between this fungal group and its plant host is mutualistic. The combination of AMF and DSE significantly and positively influenced plant above-ground growth and root morphology. These findings support the hypothesis that AMF + DSE combined inoculation has a synergistic effect in promoting the growth of the host plant at the low DSE density and a competitive effect at medium and high DSE densities. Treatments AM + MD and AM + HD exerted the greatest effects on host plant growth and root morphology. Future studies investigating the allocation of nutrient resources between both fungal groups and plants would increase our understanding of dual interactions.

## Methods

### Isolation and identification of DSE

Roots of *Stipa krylovii* were surface-sterilized in 75% ethanol and 5% sodium hypochlorite for 5 min, rinsed three times in deionized water, and then transferred to potato dextrose agar (PDA) culture medium with antibiotic supplements (ampicillin and streptomycin sulfate) and incubated at 27 °C [43]. The isolated DSE was identified as *Alternaria* sp. by molecular identification and deposited in the general microbiology center of China National Committee (CGMCC, address: 3, Courtyard 1, Beichen West Road, Chaoyang District, Beijing), with the preservation number CGMCC No.17463. *Diversispora epigaea* (formerly *Glomus versiforme*) was used as the AMF experimental material.

### Preparation of AMF and DSE inocula

The DSE inoculum was prepared by aseptic growth in flasks with Modified Melin-Norkra (MMN) medium. The inoculated flasks were oscillated at 170 rpm and incubated at 27 °C in the dark for 2 weeks. The AMF *Diversispora epigaea* was provided by the Institute of Plant Nutrition and Resources, Beijing Academy of Agriculture and Forestry Sciences, and the inoculum contained spores, external mycelium and mycorrhizal root fragments with a spore density of 26 g^− 1^, a colonization rate of 87%, and a hyphal length of 3.12 m g^− 1^. Controls received DSE and AMF inocula sterilized by autoclaving at 121 °C for 30 min.

### Greenhouse experiment

Maize seeds were acquired from the Zhongnongzuo Technology Development Co. Ltd. of Beijing and surface-sterilized in 70% (v/v) ethanol for 5 min and in 10% (v/v) NaClO for 10 min, rinsed several times with deionized water and placed in a culture dish with moist sterile filter paper in the dark at 25 °C for 3 days. Three maize seeds were sown per pot and one plant was retained at the three-leaf stage. Plants were inoculated with *Alternaria* sp. or *D. epigaea* or inoculated combined (AMF inoculation × 3 DSE densities) with four replicates of each treatment. The AM treatment consisted of 50 g *D. epigaea* (1300 spores) and sterilized DSE per pot. The three DSE densities were low (LD, 2 × 10^5^ CFU mL^− 1^), medium (MD, 4 × 10^5^ CFU mL^− 1^) and high (HD, 8 × 10^5^ CFU mL^− 1^) with sterilized AMF, respectively. Combined inoculations of AMF + DSE consisted of AM (50 g) + LD (50 mL), AM (50 g) + MD (50 mL), or AM (50 g) + HD (50 mL). A control consisted of equal amounts of sterilized AMF and DSE medium. A total of 32 pots (8 treatments × 4 replicates) were grown for 60 days before harvest.

### Plant measurement and fungal infection

Plant height and ground diameter were recorded every 10 days. Gas exchange was measured using a portable photosynthesis system (Li-6400; Li-cor Inc., Lincoln, NE), comprising photosynthesis (P_n_), stomatal conductance (G_s_), transpiration rate (T_r_) and intercellular CO_2_ concentration. The relative chlorophyll content of leaves was measured with a SPAD-502 leaf chlorophyll meter (Minolta, Osaka, Japan). Plant roots and shoots were harvested and weighed fresh and dry (60 °C, 48 h), and oven-dried samples were used to determine plant nutrient (phosphorus and potassium) contents and the phytohormone gibberellic acid (GA), the hormones abscisic acid (ABA), indole-3-acetic acid (IAA), and cytokinin (CTK) were determined after 60 days. Fine roots selected from the soil were collected to determine colonization by DSE and AMF. The endogenous hormones (GA, CTK, IAA, and ABA) and fungal colonization were measured according to the methods of Bi et al. [9]. Leaf area was determined by plotting the shape of leaves on cardboard and calculating the leaf area through a square grid before drying. The contribution of fungi to biomass was evaluated in terms of inoculation responsiveness, calculated as (total biomass of inoculated maize − total biomass of non-inoculated maize) / total biomass of inoculated maize × 100%.

The general inoculation effect was evaluated by fungal total colonization and colonization intensity in the root system [44]. Each treatment was examined using the glass slide method in which 30 randomly-selected 0.5-cm-long root segments were cleared with 10% (w/v) potassium hydroxide and stained with 0.5% (w/v) acid fuchsin [45]. Fungal total infection (%) is expressed as the percentage of infected fine root segments in each root sample: infection intensity (%) = (infected length of root segments / total length of infected root segments) × 100%. Root system morphology, comprising total root length, number of root tips, root surface area, and average root diameter and volume, were evaluated using RootSnap software (CID Bio-Science, Camas WA).

### Soil properties

Soil available phosphorus (AP), available potassium (AK), total phosphorus (TP), and total potassium (TK) were determined by inductively coupled–plasma emission spectroscopy (ICP-OES, Optima 5300DV, Perkin Elmer, Waltham, MA). The total nitrogen (TN) contents were determined by the Kjeldahl method [46]. Soil phosphatase was determined by the method of Tarafdar and Marschner [47] and soil urease activity by that of Hoffmann and Teicher [48]. Soil glomalin was quantified as glomalin-related soil protein (GRSP). Easily extractable BRSPs (EE-BRSPs, EEG) and total BRSPs (T-BRSPs, TG) were determined by the methods of Wright and Upadhyaya and Janos et al. [49, 50] using bovine serum albumin as the standard to determine the GRSP concentration in the extracts by Bradford assay.

### Statistical analysis

The effects of the treatments on the measured variables were evaluated by one-way analysis of variance (*P* < 0.05) and differences between mean values by Tukey’s multiple-range test (*P* < 0.05) using the SPSS v. 21.0 for Windows software package (IBM Corp., Armonk, NY). Pearson’s correlation analysis and the structural equation model (SEM) were used to test the effects of DSE on plant growth using the SPSS software package (version 19.0, SPSS, Chicago, IL) and AMOS software (version 21.0, Amos Development Corp., Meadville, PA).

## Supplementary Information

**Additional file 1.**

## Data Availability

All data generated or analyzed during this study are included in this manuscript and its supplementary information files, and the datasets used and/or analyzed during the current study are available from the corresponding author on reasonable request.

## Abbreviations

AMF: Arbuscular mycorrhizal fungi
DSE: Dark septate endophytes
PDA: Potato dextrose agar
MMN: Modified Melin-Norkra
Pn: Photosynthesis
Gs: Stomatal conductance
Tr: Transpiration rate
IAA: Indole-3-acetic acid
CTK: Cytokinin
ABA: Hormone abscisic acid
GA: Gibberellic acid
AP: Available phosphorus
AK: Available potassium
TN: Total nitrogen
TP: Total phosphorus
TK: Total potassium
EEG: Easily extractable glomalin-related soil protein
TG: Total glomalin-related soil protein
ALP: Activity of alkaline phosphatase
U: Activity of soil urease
CK: Treatments with sterilized DSE and AMF
AM: Sterilized DSE with AMF
LD: Low concentration of DSE with sterilized AMF
MD: Medium concentration of DSE with sterilized AMF
HD: High concentration of DSE with sterilized AMF
AM + LD: Low concentration of DSE with AMF
AM + MD: Medium concentration of DSE with AMF
AM + HD: High concentration of DSE with AMF

## References

[1] Read DJ, Perez-Moreno J (2003). Mycorrhizas and nutrient cycling in ecosystems: a journey towards relevance?. New Phytol.

[2] Hardoim PR, Van Overbeek LS, Berg G, Pirttilä AM, Compant S, Campisano A, Döring M, Sessitsch A (2015). The hidden world within plants: ecological and evolutionary considerations for defining functioning of microbial endophytes. Microbiol Mol Biol Rev.

[3] Selvakumar G, Shagol CC, Kim K, Han S, Sa T (2018). Spore associated bacteria regulates maize root K+/Na+ ion homeostasis to promote salinity tolerance during arbuscular mycorrhizal symbiosis. BMC Plant Biol.

[4] Bahadur A, Batool A, Nasir F, Jiang S, Mingsen Q, Zhang Q, Pan J, Liu Y, Feng H (2019). Mechanistic insights into arbuscular mycorrhizal fungi-mediated drought stress tolerance in plants. Int J Mol Sci.

[5] Ahammed GJ, Mao Q, Yan Y, Wu M, Wang Y, Ren J, Guo P, Liu A, Chen S (2020). Role of melatonin in arbuscular mycorrhizal fungi-induced resistance to Fusarium wilt in cucumber. Phytopathology.

[6] Govindarajulu M, Pfeffer PE, Jin H, Abubaker J, Douds DD, Allen JW (2005). Nitrogen transfer in the arbuscular mycorrhizal symbiosis. Nature.

[7] de Novais CB, Sbrana C, da Conceição JE, Rouws LFM, Giovannetti M, Avio LV (2020). Mycorrhizal networks facilitate the colonization of legume roots by a symbiotic nitrogen-fixing bacterium. Mycorrhiza..

[8] Holátko J, Brtnický M, Kučerík J, Kotianova’ M, Elbl J, Kintl A (2021). Glomalin–truths, myths, and the future of this elusive soil glycoprotein. Soil Biol Biochem.

[9] Bi YL, Zhang J, Song ZH, Wang ZG, Qiu L, Hu JJ, Gong YL (2019). Arbuscular mycorrhizal fungi alleviate root damage stress induced by simulated coal mining subsidence ground fissures. Sci Total Environ.

[10] Tian L, Shi S, Ma L, Zhou X, Luo S, Zhang J (2019). The effect of *Glomus intraradices* on the physiological properties of *Panax ginseng* and on rhizospheric microbial diversity. J Ginseng Res.

[11] Wu YQ, Liu TT, He XL (2009). Mycorrhizal and dark-septate endophytic fungi under the canopies of desert plants in mu us Sandy land of China. Front Agric China.

[12] Mandyam K, Loughin T, Jumpponen A (2010). Isolation and morphological and metabolic characterization of common endophytes in annually burned tallgrass prairie. Mycologia.

[13] Li BK, He XL, He C, Chen YY, Wang XQ (2015). Spatial dynamics of dark septate endophytes and soil factors in the rhizosphere of Ammopiptanthus mongolicus in Inner Mongolia, China. Symbiosis.

[14] Xie LL, He XL, Wang K, Hou LF, Sun Q (2017). Spatial dynamics of dark septate endophytes in the roots and rhizospheres of *Hedysarum scoparium* in Northwest China and the influence of edaphic variables. Fungal Ecol.

[15] Narisawa K, Usuki F, Hashiba T (2004). Control of *Verticillium* yellows in Chinese cabbage by the dark septate endophytic fungus LtVB3. Phytopathology.

[16] Barrow J, Aaltonen R (2001). Evaluation of the internal colonization of *Atriplex canescens* (Pursh) Nutt. Roots by dark septate fungi and the influence of host physiological activity. Mycorrhiza.

[17] Muthukumar T, Senthilkumar M, Rajangam M (2006). Arbuscular mycorrhizal morphology and dark septate fungal associations in medicinal and aromatic plants of Western Ghats, southern India. Mycorrhiza.

[18] Wu LQ, Guo SX (2008). Interaction between an isolate of dark septate fungi and its host plant *Saussurea involucrata*. Mycorrhiza.

[19] Berthelot C, Chalot M, Leyval C, Blaudez D, Hodkinson TR, Doohan FM, Saunders MJ, Murphy BR (2019). From darkness to light: emergence of the mysterious dark septate endophytes in plant growth promotion and stress alleviation.

[20] Jumpponen A (2001). Dark septate endophytes: are they mycorrhizal?. Mycorrhiza.

[21] Qin Y, Pan X, Kubicek C, Druzhinina I, Chenthamara K, Labbé J (2017). Diverse plant-associated Pleosporalean fungi from saline areas: ecological tolerance and nitrogen-status dependent effects on plant growth. Front Microbiol.

[22] Li X, He C, He X, Su F, Hou L, Ren Y (2019). Dark septate endophytes improve the growth of host and non-host plants under drought stress through altered root development. Plant Soil.

[23] Narisawa K, Hambleton S, Currah RS (2007). *Heteroconium chaetospira*, a dark septate root endophyte allied to the Herpotrichiellaceae (Chaetothyriales) obtained from some forest soil samples in Canada using bait plants. Mycoscience.

[24] Zhan F, Li B, Jiang M, Qin L, Wang J, He Y (2017). Effects of a root-colonized dark septate endophyte on the glutathione metabolism in maize plants under cadmium stress. J Plant Interact.

[25] Zhu L, Li T, Wang C, Zhang X, Xu L, Xu R (2018). The effects of dark septate endophyte (DSE) inoculation on tomato seedlings under Zn and cd stress. Environ Sci Pollut Res.

[26] Valli PPS, Muthukumar T (2018). Dark septate root endophytic fungus Nectria haematococca improves tomato growth under water limiting conditions. Indian J Microbiol.

[27] Scervino JM, Gottlieb A, Silvani VA (2009). Exudates of dark septate endophyte (DSE) modulate the development of the arbuscular mycorrhizal fungus (AMF) *Gigaspora rosea*. Soil Biol Biochem.

[28] He C, Wang W, Hou J (2020). Plant performance of enhancing licorice with dual inoculating dark septate endophytes and *Trichoderma viride* mediated via effects on root development. BMC Plant Biol.

[29] Muthukumar T, Vediyappan S (2010). Comparison of arbuscular mycorrhizal and dark septate endophyte fungal associations in soils irrigated with pulp and paper mill effluent and well-water. Eur J Soil Biol.

[30] Weishampel PA, Bedford BL (2006). Wetland dicots and monocots differ in colonization by arbuscular mycorrhizal fungi and dark septate endophytes. Mycorrhiza.

[31] Huusko K, Ruotsalainen AL, Markkola AM (2017). A shift from arbuscular mycorrhizal to dark septate endophytic colonization in *Deschampsia flexuosa* roots occurs along primary successional gradient. Mycorrhiza.

[32] He C, Wang WQ, Hou JL (2019). Plant growth and soil microbial impacts of enhancing licorice with inoculating dark septate endophytes under drought stress. Front Microbiol.

[33] Berthelot C, Blaudez D, Beguiristain T, Chalot M, Leyval C (2018). Co-inoculation of Lolium perenne with Funneliformis mosseae and the dark septate endophyte Cadophora sp in a trace element-polluted soil. Mycorrhiza.

[34] Wu LQ, Lv YL, Meng ZX, Chen J, Guo SX (2010). The promoting role of an isolate of dark-septate fungus on its host plant *Saussurea involucrata* Kar. Et Kir. Mycorrhiza.

[35] Andrade-Linares DR, Grosch R, Restrepo S, Krumbein A, Franken P (2011). Effects of dark septate endophytes on tomato plant performance. Mycorrhiza.

[36] Li X, He XL, Zhou Y, Hou YT, Zuo YL (2019). Effects of dark septate endophytes on the performance of *Hedysarum scoparium* under water deficit stress. Front Plant Sci.

[37] He YM, Fan XM, Zhang GQ, Li B, Li TG, Zu YQ (2020). Effects of arbuscular mycorrhizal fungi and dark septate endophytes on maize performance and root traits under a high cadmium stress. S Afr J Bot.

[38] Liu Y, Wei XL (2019). Dark septate endophyte improves drought tolerance of *Ormosia hosiei* Hemsley and E. H. Wilson by modulating root morphology, ultrastructure, and the ratio of root hormones. Forests.

[39] Xu R, Li T, Shen M, Yang ZL, Zhao ZW (2020). Evidence for a dark septate endophyte (*Exophiala pisciphila*, H93) enhancing phosphorus absorption by maize seedlings. Plant Soil.

[40] Franzluebbers AJ, Wright SF, Stuedemann JA (2000). Soil aggregation and glomalin under pastures in the southern Piedmont USA. Soil Sci Soc Am J.

[41] Rillig MC, Maestre FT, Lamit LJ (2003). Microsite differences in fungal hyphal length, glomalin, and soil aggregate stability in semiarid Mediterranean steppes. Soil Biol Biochem.

[42] Nichols KA, Wright SF (2005). Comparison of glomalin and humic acid in eight native US soils. Soil Sci.

[43] Zhan FD, He YM, Li T, Yang YY, Toor GS, Zhao ZW (2015). Tolerance and antioxidant response of a dark septate endophyte (DSE), *Exophiala pisciphila*, to cadmium stress. Bull Environ Contam Toxicol.

[44] Giovannini L, Palla M, Agnolucci M, Avio L, Sbrana C, Turrini A, Giovannetti M (2020). Arbuscular mycorrhizal fungi and associated microbiota as plant biostimulants: research strategies for the selection of the best performing inocula. Agronomy.

[45] Phillips JM, Hayman DS (1970). Improved procedures for clearing roots and staining parasitic and vesicular-arbuscular mycorrhizal fungi for rapid assessment of infection. Trans Br Mycol Soc.

[46] Zeng Q, Jia P, Wang Y, Wang H, Li C, An S (2019). The local environment regulates biogeographic patterns of soil fungal communities on the loess plateau. Catena.

[47] Tarafdar JC, Marschner H (1994). Phosphatase activity in the rhizosphere and hyphosphere of VA mycorrhizal wheat supplied with inorganic and organic phosphorus. Soil Biol Biochem.

[48] Hoffmann GG, Teicher K (1961). A colorimetric technique for determining urease activity in soil. Dung Boden.

[49] Wright SF, Upadhyaya A (1998). A survey of soils for aggregate stability and glomalin, a glycoprotein produced by hyphae of arbuscular mycorrhizal fungi. Plant Soil.

[50] Janos DP, Garamszegi S, Beltran B (2008). Glomalin extraction and measurement. Soil Biol Biochem.

