# Supplementary material for: Combined inoculation with dark septate endophytes and arbuscular mycorrhizal fungi: synergistic or competitive growth effects on maize?
Source: BMC Plant Biol. 2021 Oct 29;21:498. doi: 10.1186/s12870-021-03267-0 (PMC8555310; doi:10.1186/s12870-021-03267-0)
Supplement: Supplementary file 1 — Additional file 1. [file 12870_2021_3267_MOESM1_ESM.zip › v-Fig.S1.docx]

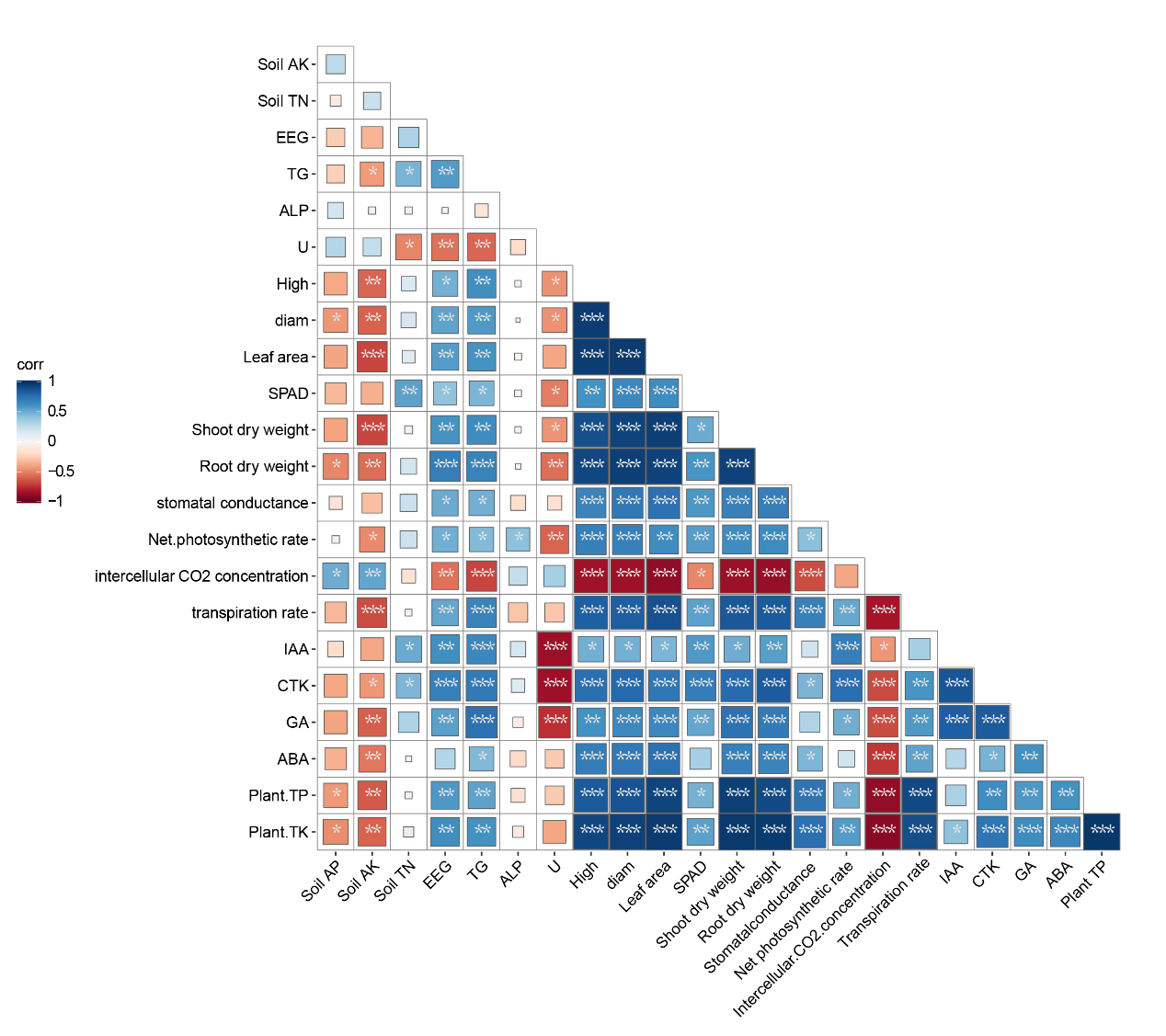


**Fig. S1** Person's correlation analysis between DSE infection intensity, plant growth and soil variables. IAA: Indole-3-acetic acid; CTK: Cytokinin; ABA: Hormone abscisic acid; GA: Gibberellic acid; AP: Available phosphorus; AK: Available potassium; TN: Total nitrogen; EEG: Easily extractable glomalin-related soil protein; TG: Total glomalin-related soil protein; ALP: Activity of alkaline phosphatase; U: Activity of soil urease. **P* < 0.05, ***P* < 0.01, ****P* < 0.001.
