# Supplementary figures and images for: Combined inoculation with dark septate endophytes and arbuscular mycorrhizal fungi: synergistic or competitive growth effects on maize?
Source: BMC Plant Biol. 2021 Oct 29;21:498. doi: 10.1186/s12870-021-03267-0 (PMC8555310; doi:10.1186/s12870-021-03267-0)

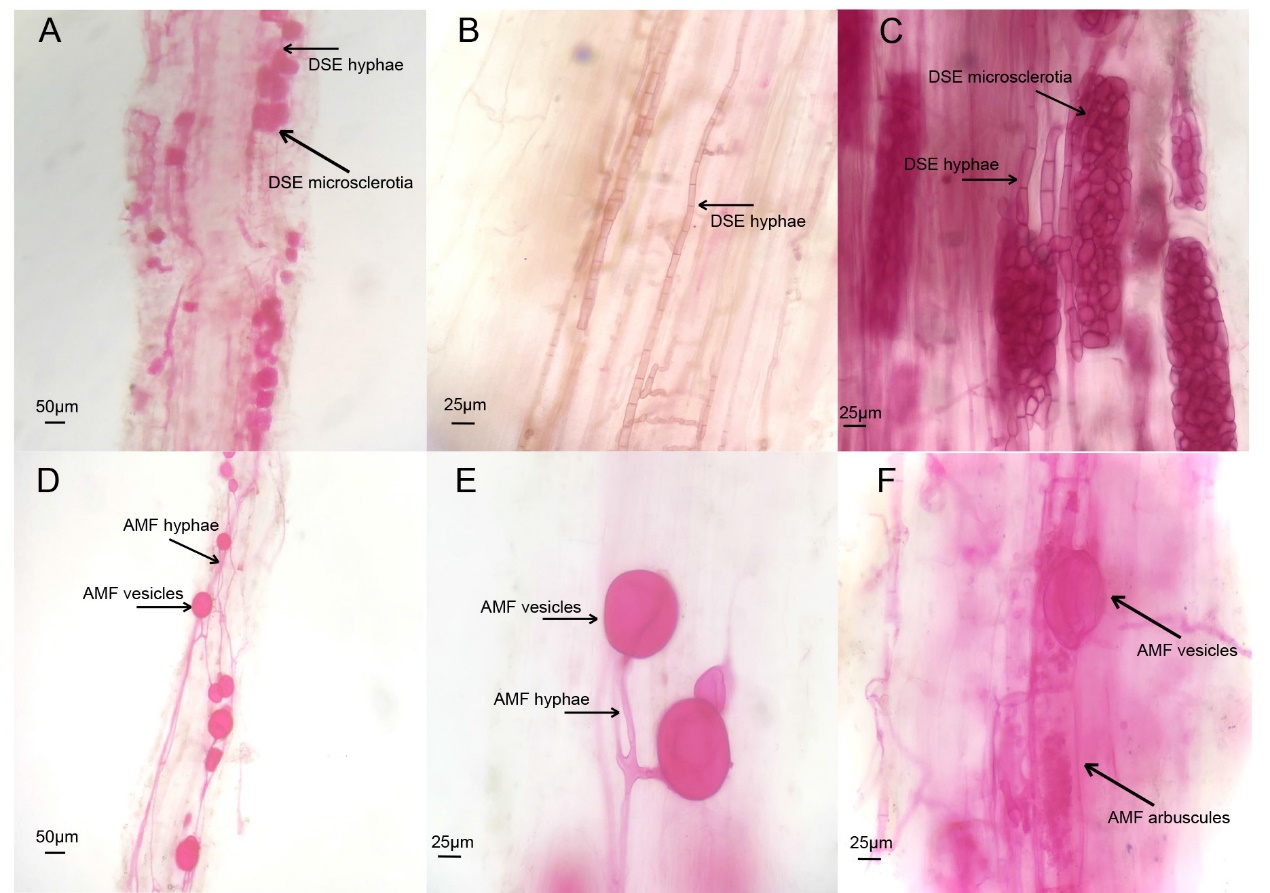


**Fig. S2** Dark septate endophytes and arbuscular mycorrhizal fungal infections in maize roots

Supplement: Supplementary file 1 — Additional file 1. [file 12870_2021_3267_MOESM1_ESM.zip › v-Fig.S2.docx]
